# Leveraging Routinely Collected Program Data to Inform Extrapolated Size Estimates for Key Populations in Namibia: Small Area Estimation Study

**DOI:** 10.2196/48963

**Published:** 2024-04-04

**Authors:** Talia Loeb, Kalai Willis, Frans Velishavo, Daniel Lee, Amrita Rao, Stefan Baral, Katherine Rucinski

**Affiliations:** 1 Data for Implementation (Data.FI) Department of Epidemiology Johns Hopkins Bloomberg School of Public Health Baltimore, MD United States; 2 IntraHealth Namibia Windhoek Namibia; 3 United States Agency for International Development Dominican Republic Santo Domingo Dominican Republic; 4 Data for Implementation (Data.FI) Department of International Health Johns Hopkins Bloomberg School of Public Health Baltimore, MD United States

**Keywords:** female sex workers, HIV, key populations, men who have sex with men, Namibia, population size estimation, small area estimation

## Abstract

**Background:**

Estimating the size of key populations, including female sex workers (FSW) and men who have sex with men (MSM), can inform planning and resource allocation for HIV programs at local and national levels. In geographic areas where direct population size estimates (PSEs) for key populations have not been collected, small area estimation (SAE) can help fill in gaps using supplemental data sources known as auxiliary data. However, routinely collected program data have not historically been used as auxiliary data to generate subnational estimates for key populations, including in Namibia.

**Objective:**

To systematically generate regional size estimates for FSW and MSM in Namibia, we used a consensus-informed estimation approach with local stakeholders that included the integration of routinely collected HIV program data provided by key populations’ HIV service providers.

**Methods:**

We used quarterly program data reported by key population implementing partners, including counts of the number of individuals accessing HIV services over time, to weight existing PSEs collected through bio-behavioral surveys using a Bayesian triangulation approach. SAEs were generated through simple imputation, stratified imputation, and multivariable Poisson regression models. We selected final estimates using an iterative qualitative ranking process with local key population implementing partners.

**Results:**

Extrapolated national estimates for FSW ranged from 4777 to 13,148 across Namibia, comprising 1.5% to 3.6% of female individuals aged between 15 and 49 years. For MSM, estimates ranged from 4611 to 10,171, comprising 0.7% to 1.5% of male individuals aged between 15 and 49 years. After the inclusion of program data as priors, the estimated proportion of FSW derived from simple imputation increased from 1.9% to 2.8%, and the proportion of MSM decreased from 1.5% to 0.75%. When stratified imputation was implemented using HIV prevalence to inform strata, the inclusion of program data increased the proportion of FSW from 2.6% to 4.0% in regions with high prevalence and decreased the proportion from 1.4% to 1.2% in regions with low prevalence. When population density was used to inform strata, the inclusion of program data also increased the proportion of FSW in high-density regions (from 1.1% to 3.4%) and decreased the proportion of MSM in all regions.

**Conclusions:**

Using SAE approaches, we combined epidemiologic and program data to generate subnational size estimates for key populations in Namibia. Overall, estimates were highly sensitive to the inclusion of program data. Program data represent a supplemental source of information that can be used to align PSEs with real-world HIV programs, particularly in regions where population-based data collection methods are challenging to implement. Future work is needed to determine how best to include and validate program data in target settings and in key population size estimation studies, ultimately bridging research with practice to support a more comprehensive HIV response.

## Introduction

There have been significant declines in HIV incidence in countries across southern Africa over the last 10 years, including in Namibia [1]. This progress largely reflects the expanded availability of comprehensive HIV prevention and treatment, including the rollout of pre-exposure prophylaxis and increased access to antiretrovirals for people living with HIV. In Namibia, approximately 95% of those living with HIV are aware of their status, 95% of those aware of their status are on treatment, and 92% of those on treatment are virally suppressed [2]. Despite these investments and progress toward the Joint United Nations Programme on HIV/AIDS (UNAIDS) 95-95-95 targets, key populations in Namibia, such as female sex workers (FSW) and gay, bisexual, and other men who have sex with men (MSM), continue to experience a disproportionate burden of HIV compared to other adults of reproductive age [3], with an estimated prevalence of 20.9% and 8.4%, respectively. Viral suppression among individuals living with HIV ranges between 31.2% and 75.5% among FSW and between 55.8% and 76.1% among MSM, with significant variation across geographic regions [2].

Key populations in Namibia face individual, network, and structural barriers to HIV prevention and treatment that increase population-level risks for HIV acquisition and transmission. Sex work is criminalized in Namibia [4], limiting the extent to which FSW are able to safely access sexual and reproductive services without the fear of stigma or penalization by law enforcement. Power imbalances within sexual relationships, high rates of sexual and physical violence, and a lack of social protections for sex workers can compromise sexual autonomy and increase the risk of HIV [5,6]. For MSM, laws that criminalize same-sex relationships between men and other intersectional stigmas have been found to increase depression and impact stress-response behaviors in Namibia, such as substance use, increasing the risk of acquiring HIV [7]. Further, enacted stigmas in clinical settings have been found to limit access to comprehensive health services for MSM, including prevention and treatment for HIV and other sexually transmitted infections [2,8-10].

Differentiated HIV services that are responsive to the needs of key populations can improve the overall quality of care and increase the uptake of HIV testing and treatment, but data are needed to guide the rollout and scale-up of these programs in Namibia [11]. Studies to determine population size for FSW and MSM in Namibia have historically been conducted in mostly urban settings, where community-led HIV programs are well-established. However, due to the high cost of incorporating less densely populated areas into empiric data collection efforts, population size estimates (PSEs) for key populations are lacking for the majority of the country.

Determining the number or size of key populations in Namibia may help identify existing gaps in programming and guide the prioritization of interventions aimed at addressing the transmission, prevention, and treatment of HIV [12]. Methodological approaches that use existing PSEs along with auxiliary data sources to determine the size of key populations are well-established [13-15]. However, these small area estimation (SAE) approaches typically exclude the use of program data as key inputs. Additionally, key population PSEs are often generated using multiple methods, and thus numerous and competing estimates may be produced for the same district or region [16,17]. While HIV programs frequently provide services in the same regions where size estimates are available and thus collect substantial data on the number and type of key populations accessing services, program data have not been used to systematically inform the triangulation of these size estimates [18]. By comparison, sentinel surveillance data and national HIV service delivery program data are regularly combined to inform estimates for the “general population,” but this approach is not currently used for key population-related estimation.

In this study, we used a consensus-informed approach with local stakeholders alongside routinely collected HIV program data provided by key population implementing partners to systematically generate subnational size estimates and demonstrate the use of the inclusion of program data for FSW and MSM in Namibia.

## Methods

### Direct Estimates and Sources of Auxiliary Data

Direct size estimates were generated using empiric data collection methods between 2012 and 2014 through Integrated Bio-Behavioral Surveillance Studies (IBBSS) in Zambezi, Ohangwena, Erongo, and Khomas for FSW [19] and in Karas, Oshana, Erongo, and Khomas for MSM (Table S1 in Multimedia Appendix 1) [20]. In the IBBSS, direct estimates were derived using multiple methods of size estimation, including mapping, key informant interviews, unique object identifiers, wisdom of the crowds, literature review, and stakeholder consensus [16,19]. These methods have been described previously [16,19]. Respondent-driven sampling was used to recruit all participants. Briefly, a set of 6-9 seeds from 4 study sites were selected, and each seed used coupons to refer others into the study. Recruitment continued until the desired sample size was reached [19,20]. Eligible FSW were aged 18 years or older and had received monetary payment for sex in the previous 6 months. Eligible MSM were aged 18 years or older and reported engaging in sex with other men in the previous 6 months. Both FSW and MSM were required to have resided in the study region for the last 6 months.

Auxiliary data to inform extrapolated estimates were identified in consultation with implementing partners and key stakeholders of key population programs in Namibia and were accessed through the Data.FI Consortium, a 5-year collaboration funded by the President’s Emergency Fund for AIDS Relief (PEPFAR) through the United States Agency for International Development (USAID) [21]. Specific data types included census data and geographically disaggregated population-level data such as regional literacy rates and HIV prevalence estimates. Auxiliary data sources comprised the Namibia 2011 Census [22], Namibia Population Projections calculated by the Namibia Statistics Agency [23], and the Namibia Population-Based HIV Impact Assessment (NAMPHIA) [24]. Census data and population projections were publicly available, while NAMPHIA data were obtained by request from the Centers for Disease Control and Prevention. Program data sources included indicators collected through PEPFAR’s key populations Data for Accountability, Transparency, and Impact Monitoring (DATIM) efforts [25] as well as indicators reported by the partner-led Key Populations—Strengthening Technical Assistance and Response for sustainable HIV prevention and treatment (KP-STAR) program [26].

Population denominators for direct estimates were extracted from Namibia’s Census Population Projections for the years in which data were collected in order to transform size estimates into standardized proportions. For FSW, this included regional estimates of cisgender women aged between 18 and 49 years; for MSM, this comprised adult cisgender men aged between 18 and 49 years. Age bands for each population were selected to align denominators with the age composition of the direct size estimates (numerators) collected through the initial IBBSS respondent-driven sampling survey.

Auxiliary variables identified for potential inclusion in SAE approaches included population density, employment rates, literacy proportions, and HIV prevalence. Population density, literacy, and employment rates were abstracted from the publicly available Namibia 2011 Census, while HIV prevalence estimates came from NAMPHIA (Tables S2 and S3 in Multimedia Appendix 1 provide comparisons of these characteristics across regions, stratified by sex). Variables were selected for inclusion in analyses based on their availability across on- and off-sample regions and through previously identified associations with key population size in the literature [13,14,27]. Population density and HIV prevalence were ultimately selected, given the variation in these data across regions where direct estimates were available. Program data used to inform priors included counts of the number of individuals who accessed services, reported through both DATIM and KP-STAR.

### Use of Program Data to Inform Imputations

Each method of direct size estimation produces a single estimate. Given the variation between available direct estimates within regions, we used a consensus estimation approach and a publicly available tool (ie, the consensus estimator tool) to generate a single estimate for each region where direct estimates were collected [28]. This allowed for the weighting of the IBBSS-collected direct estimates both by precision and by quality of study implementation. The methods of direct size estimation were ranked by level of confidence, with successive sampling population size estimation (SS-PSE) having the highest confidence, followed by unique object multiplier, mapping, stakeholder consensus, literature review, key informant interview, and lastly, wisdom of the crowds. This initial ranking scheme was based on the rigor and quality of methods, guided by the World Health Organization’s synthesis of these direct size estimation methods and investigator experience implementing these approaches [16,29-31].

Prior beliefs for the distribution of the derived size estimates were defined based on program data. Quarterly data between fiscal year (FY) 2020 quarter 3 and FY 2021 quarter 2 from KP-STAR and between FY 2016 quarter 4 and FY 2021 quarter 1 from DATIM were used. Program data included the number of FSW or MSM that were reached by programs during each quarter. A 2-tailed, 2-sample *t* test was performed to identify any differences in the underlying distribution between the KP-STAR and PEPFAR data. The data from PEPFAR and KP-STAR were pooled due to similarity in the underlying distribution, and the minimum, maximum, and median numbers were calculated, in addition to the SD for the pooled numbers from 2016 quarter 4 and 2021 quarter 2. These 4 values, along with the assumed log-normal distribution for population sizes, served as the prior beliefs for the consensus estimator tool in order to derive a single direct estimate for each region . Of note, the data availability limited the accessibility of consistently reported direct estimates across regions, such as missing direct estimates collected through SS-PSE for MSM in Karas and Oshana. The USAID team implemented routine data quality assessments of data from DATM and KP-STAR. This involved a random sample review of data captured in client files in comparison to the implementing partner’s client-level electronic database. A similar comparison was routinely collected at the aggregate level, comparing site-level monthly reports to the quarterly data.

Sensitivity analyses were completed to determine the extent to which adjusting the numerical level of confidence but retaining the rank order affected the weighting scheme and the resulting combined direct estimates. Multiple permutations and values for the confidence levels were assigned to assess the sensitivity of the rankings across the resulting triangulated estimates. The resulting size estimates generated from the consensus estimator were similar across the varying confidence levels as long as rank order was preserved.

### SAE Using Imputation and Regression

We conducted simple imputation, stratified imputation, and regression modeling to determine the regional proportion of female individuals aged between 18 and 49 years and the proportion of male individuals aged between 18 and 49 years that were FSW and cisgender MSM, respectively. The derived direct estimates from the consensus estimator tool were used in the simple imputation and stratified imputation approaches, whereas all the direct estimates were used in the regression approach to maximize the number of input data points.

For the imputation approaches, the consensus-generated direct estimates were divided by the total cisgender female population aged between 18 and 49 years for FSW and the total cisgender male population aged between 18 and 49 years for MSM to derive proportions. Using simple imputation, the mean of these proportions was applied to each of the regions where direct size estimates were not available. In the stratified imputation, HIV prevalence estimates reported by NAMPHIA were used as the stratification variable. Regions were categorized by whether their HIV prevalence fell above or below the median HIV prevalence of all the regions, as reported by NAMPHIA (17.85% among women and 7.45% among men of reproductive age). The mean of the proportions for each respective category was applied to each of the regions where direct size estimates were not available. In sensitivity analyses, simple and stratified imputations were also conducted using all the available direct size estimates to evaluate how the incorporation of program data as prior beliefs in the consensus estimator tool changed the results.

In a supplemental analysis, multivariable Poisson regression models were fit using the full set of available direct estimates to generate size estimate proportions for FSW and MSM. To ensure a maximum number of available inputs were available to fit the regression models, the consensus estimator tool was not used. Generalized estimating equations accounted for clustering by region, and candidate predictors included HIV prevalence, population density, literacy rate, employment, and annual projected population growth, all overall and stratified by sex. Model selection was determined using Akaike information criterion (AIC) and Bayesian information criterion (BIC) values, with the lowest values indicating the best fit.

The resulting estimated proportions from all approaches were applied to the projected 2021 population of female individuals for FSW and male individuals for MSM aged between 15 and 49 years, resulting in the final PSEs.

### Software

Imputations were conducted using Microsoft Excel [32], multivariable Poisson regression was performed using Stata (version 16) [33], and maps were constructed using ArcGIS software [34,35].

### Consensus Building With Local Stakeholders

The process of generating the extrapolated size estimates was an iterative process, requiring collaboration and discussion between researchers and government, programs, and community stakeholders in order to refine and select a final set of estimates. Meetings with implementing partners occurred on multiple occasions, with discussion around how these size estimates could be used programmatically to inform service delivery targets. A brief synopsis of these meetings has been provided in Multimedia Appendix 1.

### Ethical Considerations

This study comprised a secondary analysis of deidentified surveillance data and routinely collected programmatic data; patient-level data were not shared with the study team nor included in analyses. The Johns Hopkins Bloomberg School of Public Health Institutional Review Board designated this work as not being a human subjects research study (IRB00007442).

## Results

### Direct PSEs

Direct PSEs collected through the IBBSS varied across direct estimation methods and by region for FSW (Table 1). The largest number of FSW was generally seen in Khomas, ranging from 100 (95% CI 50-1700) using key informant interviews to 5240 (95% CI 3373-11,706) using the unique object multiplier method. Khomas also produced the largest PSEs for MSM, ranging from 300 (95% CI 100-1600) using key informant interviews to 2416 (95% CI 850-4000) using stakeholder consensus. After incorporating program data as priors in the consensus estimator tool, the resulting generated PSEs in Khomas were 1480 (95% CI 1099-1799) for FSW and 511 (95% CI 143-958) for MSM. Direct PSEs for each region, including the resulting weighted estimates from the consensus estimator tool, are presented in Table 1.

**Table 1:** Direct estimates (2012-2014 and 2019), by key population and region in Namibia. Data were collected between 2012 and 2014 and in 2019 through the Integrated Bio-Behavioral Surveillance Studies (IBBSS) studies across 4 regions in Namibia. The direct estimates collected by mapping, key informant interviews, unique object multipliers, wisdom of the crowds, literature review, stakeholder consensus, and successive sampling population size estimation (SS-PSE) were used in the consensus estimator to derive the final direct estimate included in the imputations.

Direct estimation method	Female sex worker regions, n (95% CI)					Men who have sex with men regions, n (95% CI)			
	Zambezi	Ohangwena	Erongo	Khomas	Karas		Oshana	Erongo	Khomas
Mapping	284 (142-426)	158 (79-237)	322 (161-483)	528 (264-792)	282 (141-423)		78 (39-117)	488 (244-732)	460 (230-690)
Key informant interview	300 (50-4300)	100 (30-800)	330 (200-1000)	100 (50-1700)	1132 (200-2948)		2000 (250-5184)	100 (70-300)	300 (100-1600)
Unique object multiplier	5299 (3500-8575)	1494 (1249-1822)	2352 (1597-4557)	5240 (3373-11,706)	1714 (1292-2359)		3538 (2379-5632)	2982 (2013-5808)	2229 (1699-3240)
Wisdom of the crowds	300 (100-1000)	500 (300-1000)	700 (200-2000)	600 (200-1500)	100 (40-400)		150 (50-500)	70 (40-250)	400 (no bounds)
Literature review	84 (47-251)	85 (47-254)	241 (134-723)	1582 (1055-2110)	84 (24-138)		157 (45-259)	427 (122-701)	1207 (345-1983)
Stakeholder consensus	800 (380-2000)	900 (775-2750)	900 (825-1500)	3000 (1800-3400)	500 (300-650)		500 (350-800)	610 (475-658)	2416 (850-4000)
SS-PSE	674 (318-2426)	900 (775-2750)	1057 (576-3369)	2196 (1651-2382)	—^a^		—	670 (410-1610)	2416 (850-4000)
Consensus estimator tool	208 (88-420)	302 (189-453)	387 (194-648)	1480 (1099-1779)	134 (15-294)		75 (23-147)	404 (209-562)	511 (143-958)

^a^Not available.

### Indirect Estimates

Extrapolated national estimates for FSW ranged from 4777 (stratified imputation by HIV prevalence) to 13,148 (stratified imputation by population density, preprogrammatic data). For MSM, estimates ranged from 4611 (simple imputation and stratified imputation by HIV prevalence) to 10,171 (stratified imputation by population density). Regional estimates from the imputation approaches are reported in Tables 2 and 3 for FSW and Tables 4 and 5 for MSM. Regional estimates from multivariable Poisson regression models are included in Table S4 in Multimedia Appendix 1.

**Figure 1:**
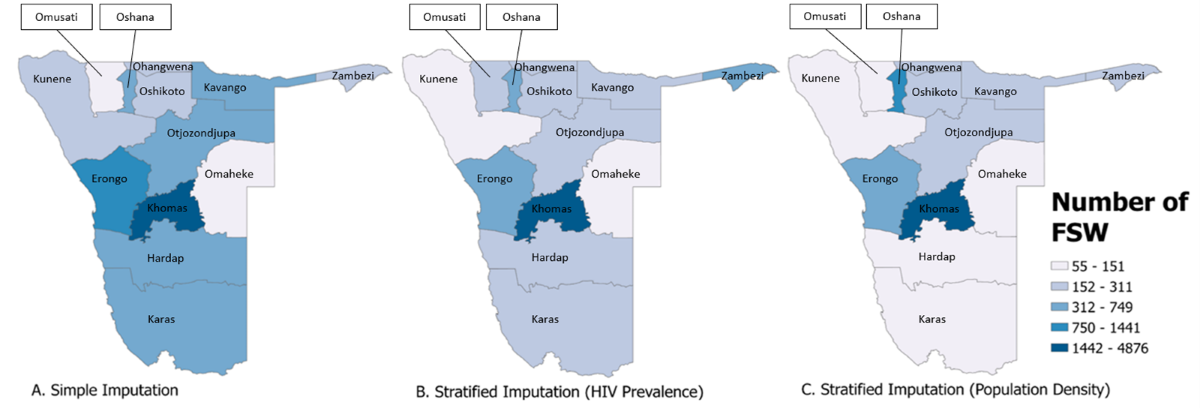
Map representing 2021 extrapolated estimates of the number of female sex workers (FSWs) across regions in Namibia. The darker gradations represent a higher number of FSWs in the region. The number of FSWs was calculated through 3 methods of imputation: simple imputation, stratified imputation (by HIV prevalence), and stratified imputation (by population density). Direct estimates used for the imputation were derived from a Bayesian consensus estimation approach incorporating routinely collected program data as priors.

**Figure 2:**
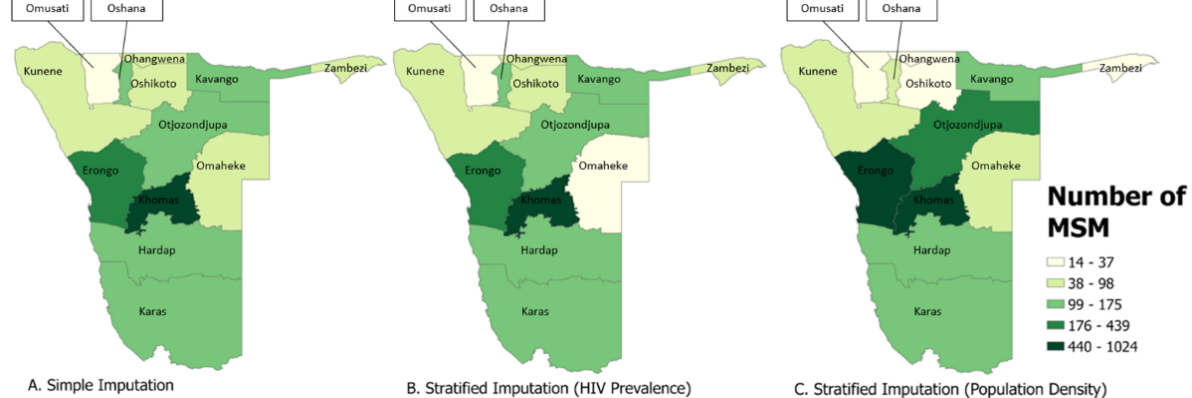
Map representing 2021 extrapolated estimates of the number of men who have sex with men (MSM) across regions in Namibia. The darker gradations represent a higher number of MSM in the region. The number of MSM was calculated through 3 methods of imputation: simple imputation, stratified imputation (by HIV prevalence), and stratified imputation (by population density). Direct estimates used for the imputation were derived from a Bayesian consensus estimation approach incorporating routinely collected program data as priors.

When program data were incorporated as priors in the imputation models, national extrapolated estimates ranged from 4777 (stratified imputation by HIV prevalence) to 9288 (simple imputation) for FSW. For MSM, these estimates ranged from 2236 (stratified imputation by population density) to 2372 (simple imputation). Regional estimates resulting from the inclusion of programmatic priors are presented by population for each approach in Tables 2-5 and are presented visually in Figures 1 and 2.

**Table 2:** Absolute estimate differences in female sex workers (FSW) extrapolated estimates for regions in Namibia before and after the integration of program data. Results from 3 different methods of imputation are presented: simple imputation, stratified imputation (by HIV prevalence), and stratified imputation (by population density).

Region	Simple imputation			Stratified imputation, HIV prevalence			Stratified imputation, population density	
	Preprogram data estimate	Postprogram data estimate	Preprogram data estimate		Postprogram data estimate	Preprogram data estimate		Postprogram data estimate
Zambezi^a^	175	256	231		404	749		311
Ohangwena^a^	129	189	171		299	680		230
Erongo^a^	985	1441	695		609	1607		523
Khomas^a^	2751	4022	1941		1701	1608		4876
Hardap	284	415	200		175	638		151
Karas	278	406	196		172	716		147
Kavango	368	538	260		227	1766		195
Kunene	140	204	99		86	698		74
Omaheke	103	151	73		64	445		55
Omusati	74	107	97		170	672		130
Oshana	512	749	361		317	619		908
Oshikoto	125	183	165		288	1897		222
Otjozondjupa	428	626	302		265	1052		227

^a^Indicates regions in which direct estimates were available.

**Table 3:** Proportion estimate differences in female sex workers (FSW) extrapolated estimates for regions in Namibia before and after the integration of program data. Results from 3 different methods of imputation are presented: simple imputation, stratified imputation (by HIV prevalence), and stratified imputation (by population density).

Stratified imputation	Simple imputation		Stratified imputation, HIV prevalence		Stratified imputation, population density	
	Preprogram data proportion	Postprogram data proportion	Preprogram data proportion	Postprogram data proportion	Preprogram data proportion	Postprogram data proportion
Greater than median	0.019	0.028	0.026	0.040	0.011	0.034
Less than or equal to median	0.019	0.028	0.014	0.012	0.028	0.010

**Table 4:** Absolute estimate differences in men who have sex with men (MSM) extrapolated estimates for regions in Namibia before and after the integration of program data. Results from 3 different methods of imputation are presented: simple imputation, stratified imputation (by HIV prevalence), and stratified imputation (by population density).

Region	Simple imputation			Stratified imputation, HIV prevalence			Stratified imputation, population density	
	Preprogram data estimate	Postprogram data estimate	Preprogram data estimate		Postprogram data estimate	Preprogram data estimate		Postprogram data estimate
Zambezi	114	59	165		63	421		35
Ohangwena	81	42	117		45	789		25
Erongo^a^	854	439	741		403	1167		612
Khomas^a^	1991	1024	1728		941	2249		621
Hardap	215	110	186		101	455		154
Karas^a^	196	100	282		109	452		140
Kavango	215	111	310		119	802		154
Kunene	96	49	84		45	437		69
Omaheke	78	40	67		37	346		56
Omusati	43	22	62		24	801		13
Oshana^a^	313	161	452		174	721		97
Oshikoto	96	49	139		53	804		30
Otjozondjupa	313	161	272		148	721		224

^a^Indicates regions in which direct estimates were available.

**Table 5:** Proportion estimate differences in men who have sex with men (MSM) extrapolated estimates for regions in Namibia before and after the integration of program data. Results from 3 different methods of imputation are presented: simple imputation, stratified imputation (by HIV prevalence), and stratified imputation (by population density).

Stratified imputation	Simple imputation		Stratified imputation, HIV prevalence		Stratified imputation, population density	
	Preprogram data proportion	Postprogram data proportion	Preprogram data proportion	Postprogram data proportion	Preprogram data proportion	Postprogram data proportion
Greater than median	0.015	0.0075	0.021	0.008	0.014	0.0045
Less than or equal to median	0.015	0.0075	0.013	0.007	0.015	0.010

### Change in Imputed Estimates Following Inclusion of Program Data

After including program data as priors in the simple imputation model for FSW, the estimated proportion of female individuals aged between 15 and 49 years who were FSW increased from 1.9% to 2.8% (Table 3). Among male individuals aged between 15 and 49 years, the proportion of MSM decreased from 1.5% to 0.75% (Table 5).

In models where HIV prevalence informed stratified imputations, the estimated proportion of women who were FSW increased from 2.6% to 4.0% in regions where HIV prevalence was greater than the median; in regions with less than median HIV prevalence, the estimated proportion decreased from 1.4% to 1.2%. Among MSM, the proportion decreased from 2.1% to 0.8% in regions where the HIV prevalence was greater than the median and decreased from 1.3% to 0.7% in regions where the HIV prevalence was less than the median.

In models where population density informed strata, the estimated proportion of women who were FSW increased from 1.1% to 3.4% in regions with a greater than median population density and decreased from 2.8% to 1.0% in regions with less than median population density. Among MSM, the proportion decreased from 1.4% to 0.45% in regions where the population density was greater than the median and decreased from 1.5% to 1.0% in regions where the population density was less than the median.

## Discussion

In this study of key population PSEs in Namibia, we calculated national and regional size estimates for FSW and MSM by incorporating routinely collected program data into established methodological approaches for SAE. These estimates were further refined by engaging implementing partners in consensus-building meetings throughout the estimation process. An iterative qualitative ranking process was used to ground truth estimates with partners. The resulting indirect size estimates varied across SAE approaches, and final estimates were calibrated to population-level data, stakeholder knowledge, and data routinely collected through HIV prevention and treatment programs in Namibia.

The inclusion of program data as previous knowledge increased the estimated proportion of reproductive-age women that were sex workers in regions with high HIV prevalence, suggesting that network-based surveys may have underestimated the true size of the sex worker population in Namibia [36]. Methods to collect direct PSEs vary in rigor and quality, but the quality of implementation and reporting of these estimates can be highly variable irrespective of method, making estimates difficult to compare [30]. Existing direct estimates for FSW from the IBBSS studies included in this analysis were also highly uneven across PSE approaches, necessitating a systematic approach to combine estimates into an interpretable and usable estimate for each region [18,36,37]. This approach may be relevant to similar settings in which direct size estimates are derived from multiple epidemiologic studies and necessitate a formalized approach for consolidation.

Historically, key population program data have not been included in size estimation exercises, given concerns around external validity. While these data are imperfect, in the case of SAEs, the number of sex workers accessing services through established key population programs can potentially serve as a lower bound for these estimates in a given region. This approach demonstrates the potential of including program data in SAE efforts to better align PSEs with real-world data in countries where surveillance efforts may be limited [14,18].

Indirect estimates for MSM were also highly sensitive to the inclusion of programmatic priors, producing smaller than expected population proportions for all regions. Despite efforts in Namibia to reach MSM with targeted HIV prevention and treatment services, including investments in peer navigators and community health workers [38], MSM remain largely marginalized from existing HIV programs relative to FSW. Provision of services to MSM remains a challenge amid Namibia’s legal framework, and as a result, the majority of MSM living with HIV are thought to be unaware of their HIV status. This is particularly true for younger MSM, who are significantly less likely to access HIV testing services relative to older MSM despite a high concentration of risk during adolescence and early adulthood [39]. While age-oriented and culturally relevant HIV testing programs may increase the uptake of HIV services for young MSM in Namibia, in the absence of larger structural interventions that reduce stigmas and discriminatory policies, these innovations may result in only modest progress in reducing the burden of HIV among MSM [40]. As size estimation is often used for allocating funding and setting targets, methods that harmonize direct PSEs with real-world data in the context of these structural barriers are needed to develop realistic targets for epidemic control. Inclusion of program data in indirect size estimation methods such as SAE could potentially allow programs to implement more informed targets for the delivery of HIV prevention and treatment services, which is ultimately one of the key purposes of population size estimation studies.

These analyses were also grounded in a systematic consensus-building approach, bridging a gap in the coordination and integration of size estimation approaches between researchers and implementing partners [41]. Meetings with key stakeholders were highly influential in the development of indirect estimates, as the limited number of data inputs challenged the precise selection of a universal best-fitting model for each population. Results for up to 3 models were presented to stakeholders to ground-truth potential estimates and serve as an additional appraisal tool. Historically, consensus-building approaches have been used to guide the triangulation of direct PSEs resulting from multiple data collection methods [42-44]. Collaborative efforts that extend beyond the initial data collection processes are seemingly less common. While ideal estimates may vary for different stakeholders, particularly when estimates are used for program target setting, this approach may provide a way forward for ensuring that size estimates are ultimately used to guide practice, programming, and resource allocation.

There are several notable strengths in this study. First, we demonstrated the potential of program data as an underused data source to understand the size of key populations in Namibia, thus opening the possibility of routinizing this approach for size estimation activities in other settings. Second, we systematically engaged with external partners to understand the cultural and on-the-ground implementation context for SAE, which provided a robust understanding of the results and interpretation. Lastly, there was a wide availability of various program and auxiliary data sources spanning multiple regions and years. This enabled the use of longitudinal data to generate prior beliefs regarding the distribution of FSW and MSM in this specific context and to use characteristics specific to each region in order to predict size estimates, which has not been done previously in Namibia.

This study also had several limitations. Despite widely available auxiliary data, there was a lack of available direct size estimates for key populations in Namibia, and those that were available were limited to more urban regions and were from the mid-2010s. As these estimates informed the primary inputs for our imputation approaches and regression modeling, it is possible that extrapolated size estimates were upwardly biased in more rural settings or in communities where outmigration is common [45]. Furthermore, the direct estimates included in analyses may have been sensitive to other unmeasured characteristics, which could have subsequently impacted the size and direction of the resulting indirect estimates. Similarly, we were only able to use programmatic data from regions where size estimates had also been collected to inform our models, as these data informed triangulation of the available direct estimates. While these data may have improved the validity of extrapolated size estimates in regions where direct estimates were available, it is possible that their inclusion introduced additional biases in regions where these data were not available [15]. Moreover, there was limited regional heterogeneity in the availability of direct estimates and program data, resulting in a lack of nuanced indirect estimates in more rural areas.

In this study of indirect PSEs in Namibia, data routinely collected by key population implementing partners were incorporated as prior information into imputation models to produce updated regional and national FSW and MSM estimates. The findings provide a potential roadmap for the systematic integration of program data in generating HIV estimates for key populations and align size estimation efforts with best practices by UNAIDS and others that rely heavily on both sentinel surveillance and national service delivery data to inform general population HIV projections. Further, as PEPFAR and other international donors move toward a model of sustainable HIV programming in sub-Saharan Africa, integrating routinely collected data into SAE approaches can support a more precise calculation of costs and allow for an expanded evidence base for supporting targeted programmatic scale up. Finally, as funding and resources for large epidemiologic studies become increasingly scarce, harnessing underused data sources, such as routinely collected programmatic data, will be critical for improving the validity of PSEs and meeting the needs of key populations in Namibia and elsewhere.

## Appendix

Multimedia Appendix 1 Supplemental materials include description of consensus building, example determination of confidence values, multivariable regression-derived populations size estimates by region, and regional characteristics stratified by sex.

## Abbreviations

AIC: Akaike information criterion
BIC: Bayesian information criterion
DATIM: Data for Accountability, Transparency, and Impact Monitoring
FSW: female sex workers
FY: fiscal year
IBBSS: Integrated Bio-Behavioral Surveillance Studies
KP-STAR: Key Populations—Strengthening Technical Assistance and Response for sustainable HIV prevention and treatment
MSM: men who have sex with men
NAMPHIA: Namibia Population-Based HIV Impact Assessment
PEPFAR: President’s Emergency Fund for AIDS Relief
PSE: population size estimate
SAE: small area estimation
SS-PSE: successive sampling population size estimation
UNAIDS: Joint United Nations Programme on HIV/AIDS
USAID: United States Agency for International Development

## Data Availability

The data sets analyzed during this study are available from the corresponding author upon reasonable request.
