# Supplementary material for: Leveraging Routinely Collected Program Data to Inform Extrapolated Size Estimates for Key Populations in Namibia: Small Area Estimation Study
Source: JMIR Public Health Surveill. 2024 Apr 4;10:e48963. doi: 10.2196/48963 (PMC11027056; doi:10.2196/48963)
Supplement: Multimedia Appendix 1 [file publichealth_v10i1e48963_app1.docx]

## **Appendix**

In total, between July 2021 and January 2022, seven meetings were held with various stakeholders, including teams from Data.FI, USAID Namibia, KP-STAR, and IntraHealth Namibia.

Consensus building meetings were structured with presentations of the most recent iteration of the estimation approaches and results, followed by guided discussion led by the research team. Implementing partners had opportunities to ask clarifying questions about the methods and results, while also providing inputs regarding how the estimates aligned with their real-world implementation experience and geospatial knowledge. Once the final estimates were calculated, implementing partners were presented with a survey to determine which estimates and methodological approaches qualitatively aligned with their current understanding of FSW and MSM population sizes in Namibia. Ultimately, these discussions were used to refine and, in part, to validate the final estimates.

The initial meeting in July 2021 consisted of the discussion of the purpose of population size estimation and how data would be used. The timeline was agreed upon and benchmarks were set. The next meeting took place in August 2021 and consisted of the team presenting the small area estimation methods, as well as the process for data collation and quality assessment. Additionally, the data synthesis was reviewed and data sharing agreements for IBBS were discussed. The next meeting involved key stakeholders and implementing partners, in which methods and utility of small area estimation was presented, as was the timeline. A webinar was conducted a week later conveying the same information, while feedback was given regarding the feasibility of high PSE results and the possibility of adjusting for certain confounding variables or reassessing population denominators.

The next meeting with USAID Namibia discussed the data collected from KP-STAR and the iterative process in order to refine region-specific estimates. Following these discussions and webinars with implementing partners, a new feedback session was scheduled to review extrapolated size estimates and to assess the methods and assumptions for validity in Namibia, by region. Most questions concerned which method should be chosen and how these estimates should be used. A survey was sent out following this meeting to determine which method’s estimates seemed most appropriate. In January 2022, the new extrapolated estimates were reviewed and assumptions were again discussed. In all, the consensus building process ran from July 2021 through January 2022, each resulting in triangulation of feedback from implementing partners.

**Table S1.** Example determination of confidence values, FSW in Zambezi.

|  | **Design Confidence** | | |
| --- | --- | --- | --- |
| **Method** | **Test 1** | **Test 2** | **Test 3** |
| Mapping | 75 | 75 | 85 |
| Key Informant Interview | 55 | 60 | 65 |
| Unique Object Multiplier | 85 | 85 | 95 |
| Wisdom of the Crowd | 40 | 40 | 50 |
| Literature Review | 60 | 60 | 70 |
| Stakeholder Consensus | 65 | 60 | 75 |
| SS-PSE | 90 | 90 | 99.9 |
|  | **Results from Consensus Estimator Tool** | | |
|  | **Test 1** | **Test 2** | **Test 3** |
| **Median (SD)** | 429.29 (90.91) | 425.34 (92.66) | 367.62 (66.58) |

**Table S2.** Regional characteristics, female population.

| Region | Population Density (persons per sq. km) | Urban Female Population (%) | Female Population Size, Age 15-49 | HIV Prevalence (%) | Literacy Rate (Persons aged 15+) (%) | Employed (%) | Annual Projected Population Growth (%) |
| --- | --- | --- | --- | --- | --- | --- | --- |
| Zambezi | 6.2 | 54 | 27385 | 28 | 80.8 | 56.2 | 1.3 |
| Ohangwena | 22.9 | 54.5 | 55796 | 21.6 | 96.6 | 55.1 | 0.7 |
| Erongo | 2.4 | 47.4 | 57674 | 14.1 | 96.9 | 21.9 | 3.4 |
| Khomas | 9.3 | 50.7 | 147863 | 9.1 | 97.6 | 63.8 | 3.1 |
| Hardap | 0.7 | 51.3 | 23189 | 11.6 | 91.30 | 54.0 | 1.5 |
| Karas | 0.5 | 50.3 | 25827 | 9.5 | 96.70 | 58.6 | 1.1 |
| Kavango | 4.6 | 54.0 | 64865 | 16.5 | 74.90 | 45.6 | 1.0 |
| Kunene | 0.8 | 52.9 | 25693 | 7.5 | 63.00 | 57.2 | 2.3 |
| Omaheke | 0.8 | 52.2 | 16377 | 8.8 | 73.10 | 48.3 | 0.5 |
| Omusati | 9.1 | 56.5 | 64673 | 21.2 | 86.50 | 56.0 | 0.6 |
| Oshana | 20.4 | 54.6 | 58421 | 16.1 | 95.20 | 59.7 | 0.9 |
| Oshikoto | 4.7 | 50.7 | 51030 | 20.2 | 89.00 | 55.1 | 1.2 |
| Otjozondjupa | 1.4 | 51.6 | 38612 | 8.7 | 82.90 | 50.5 | 0.6 |

**Table S3.** Regional characteristics, male population.

| Region | Population Density (persons per sq. km) | Urban Male Population (%) | Male Population Size, Age 18+ | HIV Prevalence (%) | Literacy Rate (Persons aged 15+) (%) | Employment (%) | Annual Projected Population Growth (%) |
| --- | --- | --- | --- | --- | --- | --- | --- |
| Zambezi | 6.2 | 46.0 | 28115 | 12.1 | 87 | 68.1 | 1.3 |
| Ohangwena | 22.9 | 45.5 | 55796 | 10.1 | 87.5 | 59.6 | 0.7 |
| Erongo | 2.4 | 52.6 | 77909 | 5.4 | 96.4 | 32.2 | 3.4 |
| Khomas | 9.3 | 49.3 | 158888 | 6.4 | 87 | 75.7 | 3.1 |
| Hardap | 0.7 | 48.7 | 30406 | 6.8 | 90.8 | 73.5 | 1.5 |
| Karas | 0.4 | 49.7 | 30196 | 8.5 | 96.5 | 75.5 | 1.1 |
| Kavango | 4.6 | 46.0 | 53507 | 8.6 | 80.2 | 55.4 | 1.0 |
| Kunene | 0.8 | 47.1 | 29180 | 5.7 | 66.8 | 70.6 | 2.3 |
| Omaheke | 0.8 | 47.8 | 23122 | 6.5 | 73.5 | 69.9 | 0.5 |
| Omusati | 9.1 | 43.5 | 56590 | 8.9 | 89 | 60.8 | 0.6 |
| Oshana | 20.4 | 45.4 | 50990 | 10.8 | 96 | 66.9 | 0.9 |
| Oshikoto | 4.7 | 49.3 | 53650 | 11.7 | 87 | 64.9 | 1.2 |
| Otjozondjupa | 1.4 | 48.4 | 48122 | 6.4 | 83.4 | 73.5 | 0.6 |

**Table S4.** FSW and MSM multivariable regression-derived population size estimates, by region*.

| **Region** | **FSW** | | **MSM** | |
| --- | --- | --- | --- | --- |
|  | **Proportion** | **PSE** | **Proportion** | **PSE** |
| Zambezi | 0.0344 | 902.4 | 0.0295 | 232.9 |
| Ohangwena | 0.0106 | 661.4 | 0.0204 | 114.4 |
| Erongo | 0.0260 | 1461.9 | 0.0056 | 329.6 |
| Khomas | 0.0095 | 1395.7 | 0.008 | 1089.8 |
| Hardap | 0.0434 | 969.6 | 0.009 | 133.3 |
| Karas | 0.0331 | 829.8 | 0.0143 | 192.2 |
| Kavango | 0.0128 | 791.1 | 0.0146 | 216.7 |
| Kunene | 0.0491 | 1201.5 | 0.0063 | 41.8 |
| Omaheke | 0.0359 | 558.7 | 0.0082 | 44.2 |
| Omusati | 0.0100 | 612.2 | 0.0157 | 46.6 |
| Oshana | 0.0133 | 750.3 | 0.0234 | 503.7 |
| Oshikoto | 0.0130 | 866.9 | 0.0276 | 182.8 |
| Otjozondjupa | 0.0166 | 612.2 | 0.008 | 171.5 |
